# The Relationship Between Gut Microbiome and Ophthalmologic Diseases: A Comprehensive Review

**DOI:** 10.7759/cureus.66808

**Published:** 2024-08-13

**Authors:** Jesus Lima Barrientos, Anahi Rojas Huerta, Angel Perez Mendoza, Barbara A Abreu Lopez, Vanessa Pamela Salolin Vargas, Oxiris Yexalen Garcia Gonzalez, Mauricio A Saldaña Ruiz, Edna Diarte, Angela Juliet Torijano Sarria

**Affiliations:** 1 General Practice, Benemérita Universidad Autónoma de Puebla, Puebla, MEX; 2 General Practice, Universidad de Carabobo, Valencia, VEN; 3 General Practice, Universidad Westhill, Mexico City, MEX; 4 General Practice, Monterrey Institute of Technology and Higher Education, Monterrey, MEX; 5 Gastroenterology, Hospital Universitario Dr. José Eleuterio González, Monterrey, MEX; 6 Medicine, Universidad Autónoma de Sinaloa, Culiacan, MEX; 7 General Practice, Universidad Santiago de Cali, Cali, COL

**Keywords:** gut-eye axis, ocular diseases, gut microbiota dysbiosis, ophthalmologic disorders, gut microbiome

## Abstract

The gut microbiome has been studied in recent years due to its association with various pathological pathways involved in different diseases, caused by its structure, function, and diversity alteration. The knowledge of this mechanism has generated interest in the investigation of its relationship with ophthalmologic diseases. Recent studies infer the existence of a gut-eye microbiota axis, influenced by the intestinal barrier, the blood-retina barrier, and the immune privilege of the eye. A common denominator among ophthalmologic diseases that have been related to this axis is inflammation, which is perpetuated by dysbiosis, causing an alteration of the intestinal barrier leading to increased permeability and, in turn, the release of components such as lipopolysaccharides (LPS), trimethylamine oxide (TMAO), and bacterial translocation. Some theories explain that depending on how the microbiome is composed, a different type of T cells will be activated, while others say that some bacteria can pre-activate T cells that mimic ocular structures and intestinal permeability that allow leakage of metabolites into the circulation. In addition, therapies such as probiotics, diet, and fecal microbiota transplantation (FMT) have been shown to favor the presence of a balanced population of microorganisms that limit inflammation and, in turn, generate a beneficial effect in these eye pathologies. This review aims to analyze how the intestinal microbiome influences various ocular pathologies based on microbial composition and pathological mechanisms, which may provide a better understanding of the diseases and their therapeutic potential.

## Introduction and background

The microbiome denotes the entirety of microorganisms residing naturally within a specific environment, profoundly influencing physiological processes and overall health. Over the past two decades, advancements in microbiome research have accelerated due to techniques such as shotgun sequencing, which analyzes the entire genetic material of microbial communities; metatranscriptomics, which studies RNA transcripts to understand gene expression; and metaproteomics, which examines proteins to reveal metabolic processes and interactions. These methodologies enable in-depth analysis of microbial communities and their functions without the need for culturing, revealing critical insights into human health and disease mechanisms [1]. Exploring the microbiome's role in human health has transformed scientific inquiry, initially focused on cataloging microbial diversity; contemporary microbiome research has rapidly evolved toward elucidating functional dynamics and host interactions. This paradigm shift underscores profound advancements in understanding microbial mechanisms and their implications for disease pathogenesis and therapeutic strategies [2].

Dysbiosis, an imbalance in microbiota, is linked to inflammatory diseases through the disturbance of metabolic activities, the production of vital metabolites, and the regulation of immune responses, highlighting the microbiome's role in health maintenance and disease prevention [3]. There is already evidence that the microbiome plays a crucial role in ocular health, influencing physiological functions and interactions that contribute to maintaining ocular homeostasis and could offer avenues for therapeutic modulation in ocular diseases. Within specialized niches, the ocular microbiome itself also plays a crucial role in maintaining ocular health by contributing to immune regulation and protecting against pathogens [4-6]. Studies underscore the microbiome's influence on systemic immune responses in distant organs with mechanisms that include microbial translocation and metabolic by-products, highlighting the microbiome's pivotal role in shaping overall immune function [7]. Recent research using advanced technologies reveals the gut microbiota's significant role beyond gastrointestinal health, impacting ocular diseases such as autoimmune uveitis, retinopathies, dry eye, and glaucoma. Dysbiosis in Firmicutes and Bacteroidetes correlates with systemic inflammation and immune dysregulation in ocular pathology, suggesting potential therapeutic strategies through probiotics and dietary interventions [8].

This review explores the intricate relationship between the gut microbiome and diverse ocular diseases, focusing notably on retinal disorders alongside conditions such as uveitis, glaucoma, and dry eye.

## Review

Gut microbiome and retinal diseases

Alterations in the microbiome of patients with retinopathies such as age-related macular degeneration (AMD), diabetic retinopathy (DR), and some retinal dystrophies (RD) suggest the existence of a gut-retina axis. The axis is a novel concept whose dynamics are explained by the interaction between the blood-retinal barrier (BRB) and the intestinal barrier, derived from several factors such as dysbiosis and inflammatory response in the presence of ocular disease, which increases permeability, allowing excessive translocation of bacteria and intestinal components. Despite these known mechanisms, there are still areas of opportunity to determine how the relationship between the microbiome and different retinal pathologies works ​[9]​.

Age-Related Macular Degeneration

Age-related macular degeneration (AMD) is an acquired degenerative retinopathy that causes central vision impairment resulting from a conjunction of neovascular and non-neovascular changes ​[10]. The microbiome's role in this disease's pathophysiology has been analyzed in a study of patients with new-onset neovascular AMD, and age- and sex-matched controls without AMD showed enrichment in species of the genera *Anaerotruncus*, *Oscillibacter*, *Ruminococcus torques*, and *Eubacterium* entries and a reduction in *Bacteroides eggerthii* ​[11,12]. On the other hand, another investigation highlights that subjects with AMD present an increased number of the genera *Prevotella*, *Holdemanella*, and *Desulfovibrio*, along with a decrease in the genera *Dorea*, *Blautia*, and *Oscillospira*. In addition, a metagenomic analysis performed on these patients implicated 72 important microbial pathways in AMD patients, showing enrichment in carotenoid biosynthesis, lipid metabolism, fatty acid biosynthesis, and bacterial chemotaxis ​[13]​.

The best-validated supplementation therapy for AMD treatment contains vitamins C and E, antioxidants, beta-carotene, copper, and zinc. The microbiome's composition may influence the bioavailability of these components. In addition, the production of some antioxidants, such as indole-3-propionic acid, is entirely dependent on the intestinal microflora, and zinc absorption can be influenced by zinc competition between the microbiomes of a host's gastrointestinal tract ​[14].

Diabetic Retinopathy

Diabetic retinopathy (DR) is one of the most prevalent metabolic and blinding eye diseases. Not all diabetics develop DR, which can be explained by individual factors such as the dysregulation of the intestinal microbiome in the human body, as it could trigger the development of inflammatory, metabolic, mental, and immune diseases. It is important to mention that hyperglycemia favors inflammation due to the increase in gram-negative bacteria that increase the production of lipopolysaccharides (LPS). LPS act because they can cross the blood-retinal barrier and induce imminent responses directly in retinal cells through Toll-like receptors 4 (TLR4), leading to an inflammatory response [15,16].

A Mendelian randomization study found that the prevalence of Ruminococcaceae and the eubacterium rectale group were risk factors for DR. Another alteration in the microbiota population was a decrease in *Faecalibacterium*, *Bifidobacterium*, and *Lactobacillus* in those who developed these complications and an increase in taxa such as *Klebsiella*. *Faecalibacterium* and *Lactobacillus* were inversely related to inflammation, hyperglycemia, and insulin resistance. It has also been observed that the consumption of probiotics containing *Bifidobacterium* and *Lactobacillus* decreases blood glucose in aids and glycosylated hemoglobin in patients with diabetes mellitus, so it is concluded that these genera, as they decrease, increase the risk of microvascular complications of the disease​. These taxa could support the development of biomarkers that generate new treatments and prevention methods for DR ​[17]​.

When intestinal dysregulation exists, probiotic/prebiotic supplementation and fecal transplantation promote intestinal homeostasis. Another alternative is intermittent fasting, which is associated with a decrease in retinal complications by promoting increased production of tauroursodeoxycholic acid, which activates the Takeda G5 protein receptor as a retina neuroprotectant. This indicates that the maintenance of the intestinal microbiome has a protective effect on the retina, preventing the development of retinopathy [9].

Retinopathy of Prematurity

Retinopathy of prematurity (ROP) is an important cause of infant blindness, which is related to the intestinal microbiota among other factors. In premature infants, the microbiota can be altered and generate a dysbiosis that can lead to increased intestinal permeability and systemic inflammation. The flora of preterm neonates, as opposed to term neonates, is usually associated with an increase in *Enterococcus* and *Staphylococcus*, and a decrease in *Bacteroides* and *Bifidobacterium*, which is associated with intestinal infections and diseases [18,19].

The mechanism of this pathology is a negative regulation of vascular endothelial growth factor (VEGF) that is aggravated by a loss of maternal insulin-like growth factor (IGF-1), resulting in incomplete retinal vascularization. This alteration of IGF-1 is linked to an increase of Enterobacteriaceae species and *Staphylococcus* species that have been shown to interfere with vascular revascularization and repair [20]. Inflammation is also associated with ROP, which results in high short-chain fatty acid (SCFA) levels. SCFA results from the metabolism of certain intestinal bacteria [21]. Neonatal intermittent hypoxia (NIH), another risk factor, can generate oxidative stress and cause damage to the retina and intestinal dysbiosis, such as the depletion of *Lactobacillus*. This probiotic has been shown to have protective effects in retinopathy [18].

Inherited Retinal Dystrophies

Retinal dystrophies (RD) are a group of inherited disorders resulting from gene variants that play a fundamental role in retinal cell development, function, and maintenance [22]. The literature on the composition of the gut microbiota in RD is scarce, so much so that of the entire spectrum that exists, there are only studies related to retinitis pigmentosa (RP) and Leber congenital amaurosis (LCA) [23]. RP can cause retinal degeneration and decreased vision, progressing to total blindness [24]. One study has analyzed the connection between RP and the gut microbiome composition using a mouse model of RP with retinal degeneration 10 (rd10) due to a nonsense mutation in the phosphodiesterase 6B (*Pde6b*) gene, which was compared to wild-type control mice. Differences in four species usually present in the healthy gut microbiome were observed, which were absent in rd10 mice (Rikenella, Muribaculaceae, Prevotellaceae UCG-011, and Bacilli species). At the same time, *Bacteroides caecimuris* was overrepresented in mice with RP but was absent in controls [25]. In other ways, mutations in the *CRB1* gene can lead to the development of LCA, the earliest and most severe hereditary DR [26]. A study evaluating the relationship between the microbiome and mutated *CRB1* gene demonstrated that the colon's outer blood-retinal barrier (BRB) and intestinal epithelial barrier are significantly impaired in mice carrying a *CRB1* mutation. Disruption of permeable gut and retina leads to gut flora translocation from the lower gastrointestinal tract to the retina, resulting in bacterial-dependent retinal degeneration. Furthermore, it was shown that systemic bacterial depletion by the reintroduction of an LCA mouse model into an FG environment or after treatment with broad-spectrum antibiotics can successfully control this retinal damage [27].

Gut microbiome and other ocular diseases

*Uveitis*

Uveitis encompasses a heterogeneous group of intraocular inflammatory diseases consisting of inflammation of the uveal tract that can affect adjacent structures such as the retina or the optic nerve. It has been classified anatomically (anterior versus posterior), etiologically (infectious versus non-infectious), and pathogenically (autoimmune versus autoinflammatory). Autoimmune (non-infectious) uveitis represents a group that frequently manifests as anterior uveitis and is part of a systemic autoimmune syndrome affecting organs other than the eye, such as human leukocyte antigen (HLA)-B27-positive spondyloarthropathies, systemic sarcoidosis, Behçet's disease, and Vogt-Koyanagi-Harada disease [28].

Until now, no clinical studies have analyzed the direct relationship between uveitis and the microbiome. However, there is evidence of the association in autoimmune diseases that present uveitis within their clinical manifestations. In general, the immune dysregulation of these pathologies induces inflammation and tissue damage, a product of an aberrant activity of T cells, which may be modulated by the altered microbiome of these patients who have in common the depletion of butyrate-producing bacteria (BPB) and methanogens that lead to an altered intestinal barrier that allows the transference of immunostimulatory factors [28-31].

In the case of Behçet's disease, there is an increase in sulfate-reducing bacteria, *Stenotrophomonas* species, *Actinomyces* species, and *Paraprevotella* species [30]. On the other hand, in Vogt-Koyanagi-Harada disease, a type of panuveitis, patients present a mix of *Bacteroides* enterotype enriched with gram-negative bacteria, such as *Bacteroides* species, *Paraprevotella* species, *Prevotella* species, and *Parabacteroides* species, accompanied by a decrease in lactate-producing bacteria [31]. Another group of diseases that have been studied is HLA-B27 positive, of which spondyloarthropathies and inflammatory bowel disease stand out, whose inflammatory pathophysiological basis allows the translocation of bacteria and microbial products to the regional lymph nodes, as is the case of components of the cell walls of gram-negative bacteria, which binds to the Toll-like receptor 4, whose expression is increased in patients with active anterior uveitis. Another example of this is microbial peptides, such as those from *Chlamydia trachomatis*, which are antigens that bind to HLA-B27, which can, in turn, induce an immune response in target organs, resulting in uveitis [29].

Potential treatments for autoimmune uveitis with the microbiome as a therapeutic target include correcting intestinal dysbiosis with probiotics that supplement good immunosuppressive bacteria and antibiotics that eliminate bad proinflammatory bacteria. Complete restoration of the intestinal microbiome by fecal microbial transplantation is also possible. Another therapeutic possibility is the administration of bacterial metabolites, such as short-chain fatty acid (SCFA), to promote the expression of regulatory T cells and increase intestinal integrity. However, none of these treatments have yet been approved and are still under investigation [32].

*Glaucoma* 

Glaucoma is a chronic progressive optic neuropathy involving retinal ganglion cells and is the leading cause of irreversible blindness worldwide. The risk factors for disease progression include intraocular pressure (IOP), vascular perfusion abnormalities, neurodegeneration, chronic inflammation, and oxidative stress [33].

Recently, a study has proposed that the intestinal microbiota plays an important role in developing glaucoma due to dysbiosis [34]. Such is the case of the untargeted association analysis of the intestinal microbiota within a British cohort, where it was found that glaucoma was negatively associated with the Mollicutes class, which was also negatively related to inflammatory bowel disease [35]. Furthermore, a study conducted in China on a cohort of patients with primary open-angle glaucoma (POAG) confirmed the correlation between gut microbiota and glaucoma. When comparing the composition of the gut microbiota in the cohort (30 patients with POAG and 30 healthy controls without POAG), it was found that Prevotellaceae, *Escherichia coli*, and Enterobacteriaceae were increased in patients with POAG. On the other hand, *Megamonas* and *Bacteroides plebeius* decreased [36].

In addition, the production of microbial metabolites such as trimethylamine (TMA) in the aqueous humor, but not betaine and trimethylamine oxide (TMAO), has been observed to be increased in patients with POAG. Bacterial toxins activate Toll-like receptor 4 (TLR4), an interconnected pathway shared between the ocular and intestinal microbiota. The binding of LPS to TLR4 activates signaling cascades that result in the production of innate effector responses and the initiation of an adaptive immune response [37].

Existing studies are still insufficient to determine the exact links between the intestinal microbiota and glaucoma, but promising results encourage further research in this area.

Dry Eye Disease 

Dry eye is a chronic ocular surface disease caused by tear film instability and imbalances in the ocular surface microenvironment that result in various symptoms of discomfort and visual problems. It can be classified according to its etiology into primary dry eye and secondary dry eye. The pathophysiological mechanisms are not fully understood; however, it is known that an inflammatory vicious circle of the ocular surface is involved, together with a dysfunction of the meibomian glands, all influenced by various local and environmental factors [38]. Intriguingly, alterations in intestinal commensal bacteria can affect the immune status of the ocular surface as an imbalance in intestinal homeostasis occurs, which can cause pathogenic microorganisms to cross the intestinal mucosal barrier, releasing inflammatory factors and activating T and B lymphocytes. Lymphatic vessels transport the inflammatory by-products to distant tissues, including the ocular surface [39,40]. In the specific case of primary dry eye, there is a decreased abundance of the butyrate-producing bacterium *Faecalibacterium*, as well as reduced levels of Treg-inducing *Clostridiales* and *Bacteroides*, which play a role in suppressing the inflammatory response in Th17 cells [41]. Regarding dry eye secondary to Sjögren's syndrome, a decrease in the *Firmicutes*/*Bacteroidetes* ratio and a decrease in *Bifidobacterium* have been observed, suggesting that the disease's severity is related to the groups of bacteria involved [42].

Derived from the demonstrated influence of the microbiome on dry eye, a recent case-control study sought to determine if there is a benefit from probiotic and prebiotic consumption supported by evidence that the modulation of the gut microbiome also modulates proteins expressed by the lacrimal glands, with an increase in IL-10 and a decrease in IL-1β and IL-6. Their promising results indicate a beneficial impact of regular consumption of probiotics and prebiotics on symptoms and clinical signs of dry eye. However, future clinical studies are needed to investigate the benefits of probiotics for patients with Sjögren's syndrome further, as they were not included in the study [43,44].

Mechanisms linking the gut microbiome and ocular diseases

The processes in which the microbiome influences ocular diseases have been studied in recent times by different studies, some of which are summarized in Table 1. This has been shown to have an enormous impact on human metabolic mechanisms, especially on the immune system, as these cells are strategically located close to the host-microbiome interface ​[45,46]. The communication between the immune system and the microbiome is through synthesizing metabolites such as carbohydrates, amino acids, and bile acid metabolites, which are recognized by receptors in immune cells. As a result of this interaction, diverse responses train these cells for possible further infections with no commensal pathogens, enhancing barrier function and tolerance [47].

**Table 1:** Relevant studies that have been conducted on the role of the microbiome in ophthalmologic diseases since 2018 AMD: age-related macular degeneration, HLA: human leukocyte antigen, POAG: primary open-angle glaucoma

Authors	Study type	Disease	Findings
Peng et al. (2024) [27]	Case-control	Retinal dystrophy (Leber congenital amaurosis due to *CRB1* gene mutation)	Mechanism connecting the gut and the eye: the principle of bacterial translocation-dependent retinal degeneration has implications that go beyond inherited retinal dystrophies.
Zhao et al. (2024) [48]	Systematic review and meta-analysis	Diabetic retinopathy	Association between alterations in the gut microbiome in type 2 diabetes and the development and progression of diabetic retinopathy: this suggests that restoring the homeostasis of the gut microbiome could be a potential way to prevent or treat diabetic retinopathy.
Morandi et al. (2024) [28]	Case-control	HLA-B27-associated non-infectious anterior uveitis	The development of uveitis is influenced by functional and compositional alterations of the intestinal microbiome. An increase in specific gram-negative bacteria and lipopolysaccharides may play a role in triggering inflammation and aberrant immune response in the eye.
Zhang et al. (2023) [49]	Case-control	AMD	AMD patients had different gut microbiota compared with healthy controls, and pathophysiology might be linked to changes in gut-related metabolic pathways.
Liu et al. (2023) [50]	Mendelian randomization	AMD	The order Rhodospirillales influenced AMD risk based on the gut-retinal axis.
Mao et al. (2023) [51]	Mendelian randomization	AMD	Causal relationship with microbiome gut taxa, including the *Eubacterium oxidoreducens* group, *Faecalibacterium*, *Ruminococcaceae* UCG-011, *Anaerotruncus*, and *Candidatus Soleaferrea*. These strains have the potential to serve as novel biomarkers.
Goodman et al. (2023) [52]	Case-control	Dry eye	Differences in the composition of the gut microbiome were found in individuals with predominantly early markers of Sjögren's syndrome compared to controls.
Liu et al. (2022) [15]	Mendelian randomization	Diabetic retinopathy	There is a possible causal relationship between some taxa of the gut microbiome and diabetic retinopathy, highlighting the association of the "gut-retina" axis and offering new insights into the mechanism of diabetic retinopathy.
Tavakoli et al. (2022) [43]	Case-control	Dry eye	The application of probiotics and prebiotics could be effective in the treatment of dry eye disease and suggests a possible alternative treatment.
Kutsyr et al. (2021) [25]	Case-control	Retinal dystrophy (retinitis pigmentosa)	Alterations in morphology and function of the rd10 mouse, an animal model of retinitis pigmentosa, demonstrate for the first time that retinal degenerative changes in neuronal and glial cells that occur in RP are concomitant with relevant changes in the gut microbiome.
Gong et al. (2020) [36]	Case-control	POAG	The first study that focused on the gut microbiome profile and its association with serum metabolites in patients with POAG.
Ye et al. (2020) [31]	Cohort	Uveitis	Distinct signature of the gut microbiome in patients with Vogt-Koyanagi-Harada, and they showed an exacerbating effect of this gut microbiome in experimental autoimmune uveitis.
Kalyana Chakravarthy et al. (2018) [53]	Cohort	Uveitis	The first study that demonstrated dysbiosis in the intestinal bacterial communities of patients with uveitis compared to healthy human subjects from a South Indian population.

Evidence has indicated that an imbalance in the gut microbiome not only exerts an effect locally but also affects other locations, such as the ocular mucosa, which is known as the gut-eye axis [10,54]. One theory explains the relationship between the gastrointestinal tract and the eyes through the T-cell threshold model, which states that depending on how the microbiome is composed, a different type of T cells will be activated; other theories say that some bacteria can preactivate T cells that mimic ocular structures and intestinal permeability, allowing leakage of metabolites into the circulation. All these theories are not mutually exclusive; on the contrary, a combination of all may be the explanation for the gut-eye axis [55].

**Figure 1:**
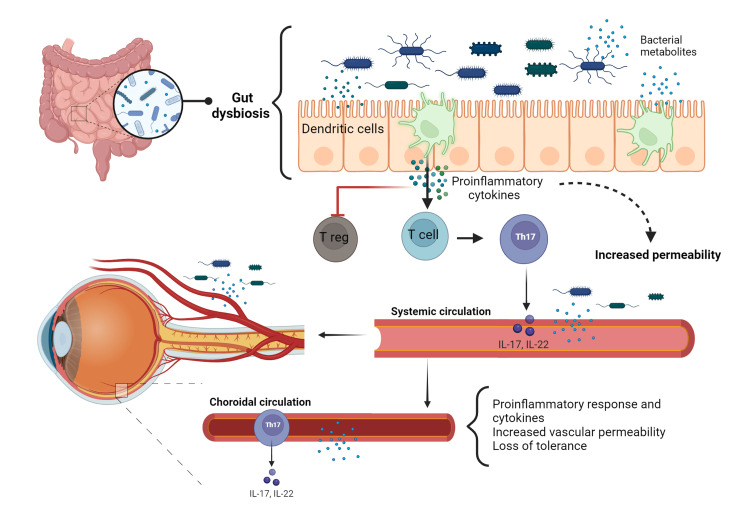
Mechanisms involved in the gut-eye axis Dysbiosis in the gut activates dendritic cells, which secretes proinflammatory cytokines (IL-6, TNF-alpha, and IL-1B), leading to the activation of specific retina T cells as proinflammatory T cells (Th17) and the inhibition of T-regulatory. Once these T cells reach the eye, they begin the secretion of proinflammatory cytokines, increased vascular permeability, loss of ocular microbiome tolerance, complement activation, and neovascularization. Also, TMAO, a metabolite produced by pathogen bacteria, is linked with the development of vascular conditions such as age-related macular degeneration; these bacteria and metabolites can travel through the systemic circulation and reach the eyes. IL-6: interleukin-6, TNF-alpha: tumor necrosis factor-alpha, IL-1B: interleukin-1B, TMAO: trimethylamine oxide, IL-17: interleukin-17, IL-22: interleukin-22 Figure created with BioRender All credits to Barbara Abreu Lopez

Dysbiosis in the gut with a high proportion of pathogenic bacteria activates dendritic cells, which secrete proinflammatory cytokines (interleukin (IL)-6, tumor necrosis factor-alpha (TNF-alpha), and IL-1B), leading to the activation of retina-specific T cells, such as proinflammatory T cells (Th17), and the inhibition of regulatory T cells. Once these T cells reach the eye, a cascade of proinflammatory processes such as secretion of proinflammatory cytokines, increased vascular permeability, loss of tolerance of the ocular microbiome, complement activation, and neovascularization is initiated [56]. Loss of intestinal barrier integrity and increased vascular permeability allow bacterial translocation and their products into the bloodstream, leading to passage across the blood-retinal barrier [57]. Once the ocular barrier has been breached, short-chain fatty acids (SCFAs) protect ocular structures by decreasing T-cell activity; however, studies have shown that trimethylamine oxide (TMAO), a metabolite produced by pathogenic bacteria associated with the development of vascular conditions such as age-related macular degeneration, can also travel through the systemic circulation and play a damaging role, inhibiting the eye's defense mechanisms (Figure 1) [58,59].

Current and potential therapeutic approaches

Probiotics and Prebiotics

Probiotics and prebiotics are increasingly known for their potential impact on the health of the gut microbiome, which extends to extraintestinal conditions. The gut microbiota plays a key role in immune modulation and the regulation of inflammation, crucial processes in the pathogenesis of previously mentioned ocular pathologies [60,61]. There is increasing evidence that the modulation of the gut microbiota by probiotics and prebiotics may be beneficial for ocular health, as they may influence the gut-eye axis, by reducing inflammation and oxidative stress [60,62,63].

Research indicates that certain strains of probiotics, such as *Lactobacillus* and *Bifidobacterium*, effectively reduce inflammation and support gut health. Combined with specific formulations, these strains have shown promise in addressing systemic inflammation, potentially benefiting ocular conditions. For instance, *Lactobacillus rhamnosus* GG has been studied for its ability to strengthen gut barrier function, which is crucial in preventing systemic inflammation that could impact the eyes [62]. Dietary interventions rich in prebiotics, such as fiber and resistant starches, promote the growth of beneficial gut bacteria and contribute to systemic anti-inflammatory effects. Prebiotics serve as food for probiotics, enhancing their effectiveness and supporting a healthy gut microbiome [63].

Impact of Diet on the Gut Microbiome and Ocular Health

Diet plays a significant role in shaping the gut microbiome. A diet high in prebiotics supports the growth of beneficial bacteria, producing short-chain fatty acids (SCFAs) that have anti-inflammatory properties. These systemic effects can help reduce inflammation in ocular tissues and improve overall eye health. To support gut and ocular health, it is recommended to increase the intake of dietary fibers from fruits, vegetables, whole grains, and fermented foods such as yogurt, kefir, and sauerkraut to provide natural probiotics. It is also recommended to reduce processed foods and sugar consumption, which can negatively affect the composition of the intestinal microbiota [63].

Fecal Microbiota Transplantation (FMT)

Fecal microbiota transplantation (FMT) involves the transfer of stool from a healthy donor to the gastrointestinal tract of a recipient. This procedure is primarily explored for gastrointestinal disorders but holds promise as a future therapeutic strategy for modulating systemic inflammation and potentially impacting ocular disease progression through microbiome alterations [64]. FMT has shown effectiveness in restoring healthy microbiota composition and reducing inflammation. Its application in ocular diseases is still under investigation, but preliminary studies suggest it could be beneficial in managing conditions such as AMD and DR by reducing systemic inflammation. The concept is based on the ability of FMT to reset the gut microbiome to a healthier state, thereby influencing systemic immune responses and potentially reducing ocular inflammation [44].

Drugs Involved in the Gut-Eye Axis 

Based on the assumption that dysbiosis is implicated in the development of ophthalmologic diseases, the proposed therapeutic strategies seek to restore the microbiota [65]. One important strategy is prebiotics and probiotics, which together provide live microorganisms beneficial to the microbiome [66]. In addition, some drugs, such as antidiabetic drugs, have been shown to have an effect on the regulation of the microbiota. Such is the case of metformin, which increases the number of goblet cells and, therefore, mucin production, improving intestinal defenses. Liraglutide and glucagon-like peptide 1 (GLP-1) agonists improve parameters related to both glucose and lipid metabolism. Others such as dipeptidyl peptidase 4 inhibitors and sodium/glucose 2 inhibitors decrease the *Firmicutes*/*Bacteroidetes* ratio, which improves the action of GLP-1, decreasing inflammatory markers and endothelial dysfunction, demonstrating a decrease in the mechanisms involved in the pathogenesis of retinopathy [65,66].

Regarding topical or intraocular ophthalmologic drugs targeting the microbiota, there is still no evidence of their existence or development, due to the newness of this line of research and the fact that the pathogenic pathways are still under study.

Future Therapeutic Strategies

**Figure 2:**
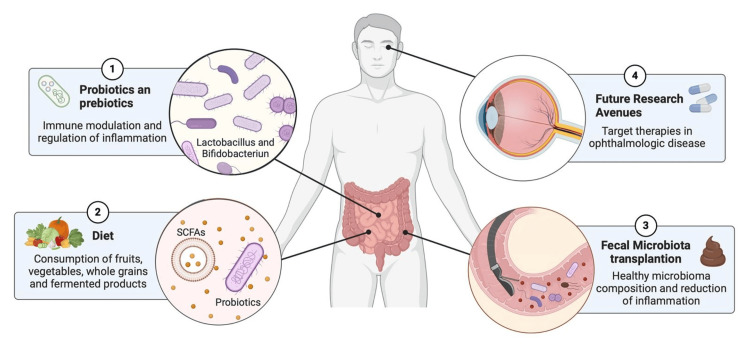
Future therapeutic strategies that may influence the gut-eye axis This diagram illustrates the benefits of therapies aimed at modifying the microbiome. Probiotics and prebiotics such as *Lactobacillus* and *Bifidobacterium* modulate the immune response and decrease inflammation. Diets rich in fermented products and fiber from fruits, vegetables, and whole grains provide natural probiotics and SCFAs that have a positive effect on the microbiota. Fecal transplantation of microbiota from a healthy donor decreases inflammation in extraintestinal diseases and may have an important role in the treatment of eye diseases. Future research into these therapies, directed at ophthalmologic diseases, represents an opportunity to provide comprehensive management of patients with these diseases. SCFAs: short-chain fatty acids Figure created with BioRender All credits to Anahi Rojas Huerta

Future therapeutic strategies may capitalize on insights into the gut-retina axis to develop targeted interventions that address the complex interplay between gut microbiota composition, systemic inflammation, and ocular health [61-63]. Ongoing research explores advanced probiotic formulations, prebiotic-rich diets, and fecal microbiota transplantation (FMT) to manage and prevent ocular diseases (Figure 2). These therapies aim to harness a healthy gut microbiome's anti-inflammatory and immune-modulating effects, as they have been previously used in other inflammatory pathologies and have shown promising results [44].

New studies are examining how specific combinations of probiotics can more effectively reduce retinal inflammation and oxidative stress. In fact, cutting-edge probiotic formulations are being customized to bolster gut barrier function, diminish systemic inflammation, and enhance metabolic health, all of which play pivotal roles in sustaining ocular health [39].

Personalized Medicine Approaches

Personalized medicine approaches leveraging individual gut microbiome profiles to tailor probiotic therapies and dietary recommendations are gaining traction. These approaches aim to optimize ocular health outcomes by targeting the gut-retina axis and its implications in disease pathogenesis. However, recent insights suggest that alterations in the ocular microbiome could disrupt ocular homeostasis and elevate susceptibility to eye infections. The frequent use of antibiotics can lead to shifts in ocular microbiota and contribute to the emergence of antibiotic-resistant strains. By tailoring interventions to the unique microbiome composition of each patient, it may be possible to manage and prevent these diseases more effectively. By understanding and harnessing these interactions, researchers and ophthalmologic clinicians aim to pave the way for novel therapeutic modalities to prevent and manage ocular diseases. This underscores the importance of medical education guiding physicians to understand these mechanisms, thereby facilitating the implementation of innovative treatments and improving clinical outcomes [67].

Limitations 

The relationship between the intestinal microbiota and its involvement in different ophthalmologic diseases is a topic of great interest. However, it is emerging, and many aspects are still not fully clarified, as is the case with specific pathogenic mechanisms. The pathways involved that have been mentioned are still not fully described, so a multiomic approach could be useful, as well as establishing the direct relationship of these mechanisms with regulatory pathways. The gut-eye axis is still under investigation, and much of the data still comes only from animal models. However, many of these show promising perspectives on the role of the microbiota both with protective effects and with a possible inflammatory association in the different regions of the eye. The microbiological agents and the different biomarkers involved in retinal diseases have not yet been fully and concretely elucidated, so the use of experimental research and applying sciences such as proteomics or sequencing may be beneficial to establish a relationship between them. In addition, the ability to identify microbiome profiles in a personalized way is still complex for current medical practice and represents a challenge in the comprehensive management of these patients. Some therapies, such as TMF, have a cautious use in clinical trials, as they are considered after failure of initial treatments, such as probiotics and prebiotics, since their metabolic impact is important and they require continuous patient monitoring.

## Conclusions

This review highlights the interrelationship between the gut microbiome and ophthalmologic diseases, starting from the concept of the gut-eye axis, influenced by the different pathological pathways discovered. Future research should prioritize elucidating more mechanisms linking alterations in the gut microbiota to ocular inflammation. Understanding these interactions could reveal new biomarkers for disease susceptibility and treatment response, facilitating personalized therapies. Collaborative efforts merging microbiological and ophthalmological expertise are crucial for translating microbiome research into clinical practice. Integrating microbiome assessments into diagnostic algorithms can revolutionize disease management by enabling early detection and targeted interventions. It is essential to explore the bidirectional relationship between ocular health and gut microbiota dynamics to provide a comprehensive framework for managing chronic diseases and associated comorbidities. Leveraging microbiome knowledge will promote greater efficacy in managing ocular diseases and thus benefit patients' quality of life.
